# Integrating neuroinformatics tools in TheVirtualBrain

**DOI:** 10.3389/fninf.2014.00036

**Published:** 2014-04-22

**Authors:** M. Marmaduke Woodman, Laurent Pezard, Lia Domide, Stuart A. Knock, Paula Sanz-Leon, Jochen Mersmann, Anthony R. McIntosh, Viktor Jirsa

**Affiliations:** ^1^Institut National de la Santé et de la Recherche Médicale UMR 1106, Institut de Neurosciences des SystèmesMarseille, France; ^2^Institut de Neurosciences des Systèmes, Aix-Marseille UniversitéMarseille, France; ^3^CodemartCluj-Napoca, Romania; ^4^CodeBox GmbHStuttgart, Germany; ^5^Rotman Research Institute at BaycrestToronto, ON, Canada

**Keywords:** Python, brain networks, connectivity, neural mass, time delays, magnetoencephalography, electroencephalography, functional MRI

## Abstract

TheVirtualBrain (TVB) is a neuroinformatics Python package representing the convergence of clinical, systems, and theoretical neuroscience in the analysis, visualization and modeling of neural and neuroimaging dynamics. TVB is composed of a flexible simulator for neural dynamics measured across scales from local populations to large-scale dynamics measured by electroencephalography (EEG), magnetoencephalography (MEG) and functional magnetic resonance imaging (fMRI), and core analytic and visualization functions, all accessible through a web browser user interface. A datatype system modeling neuroscientific data ties together these pieces with persistent data storage, based on a combination of SQL and HDF5. These datatypes combine with adapters allowing TVB to integrate other algorithms or computational systems. TVB provides infrastructure for multiple projects and multiple users, possibly participating under multiple roles. For example, a clinician might import patient data to identify several potential lesion points in the patient's connectome. A modeler, working on the same project, tests these points for viability through whole brain simulation, based on the patient's connectome, and subsequent analysis of dynamical features. TVB also drives research forward: the simulator itself represents the culmination of several simulation frameworks in the modeling literature. The availability of the numerical methods, set of neural mass models and forward solutions allows for the construction of a wide range of brain-scale simulation scenarios. This paper briefly outlines the history and motivation for TVB, describing the framework and simulator, giving usage examples in the web UI and Python scripting.

## 1. Introduction

Neuroscience has evolved through extensive interactions among disciplines to advance our appreciation of the relation between brain and behavior. The interdisciplinary nature of the field presents formidable challenges for effective collaboration.

These challenges call for two kinds of solutions. First, there is a need for comprehensive, modern computational libraries written in widely used and available programming languages; current examples include MNE-Python (Gramfort et al., 2013), a Python package for treating M/EEG data via time-frequency analyses and inverse solutions and the Brain Connectivity Toolbox (Rubinov and Sporns, 2010) for analyzing the graph theoretic properties of structural and functional connectivity. Second, there is a need for the implementation of collaborative infrastructure for sharing not only data, but expertise; CARMEN (Austin et al., 2011) and G-Node (Herz et al., 2008) are two such examples of developing platforms for collaborative work and data sharing, in the domains of cellular and systems neurophysiology.

TheVirtualBrain (TVB) provides new tools to facilitate the collaboration between experimentalists and modelers by exposing both a comprehensive simulator for brain dynamics and an integrative framework for the management, analysis, and simulation of structural and functional data in an accessible, web-based interface. The choice of Python was made based on its wide use as the high-level language in scientific programming, the unparalleled open-source libraries and tools available, and strong software engineering culture. This choice was confirmed by the publication of the first issue of Python in Neuroscience and has made it possible for the entirety of TVB from the numerical algorithms to the web server to be written in Python.

In the following, we briefly outline the scope of TVB, how to use it before detailing aspects of the architecture and simulator.

### 1.1. Overview

TVB consists of a framework and a simulator. The framework manages projects involving various data, subjects, and users and the different roles that the users might play in the projects (modeler, clinician, etc.). The framework also maintains a database of the different operations performed by the users in each project as well as the various data associated with those operations, such as structural and functional neuroimaging data. The simulator provides numerical methods to construct and simulate models based on human cortical and sub-cortical anatomy and dynamics. It employs principally a neural mass approach, incorporated realistic long-range (delayed) and short-range connectivity kernels, as well as stochastic fluctuations. To make the connection with experimental data, it provides forward solutions to compute neuroimaging data (fMRI, M/EEG) from the underlying neural dynamics. Finally, an graphical interface allows users to take advantage of the framework and simulator. These components have been described previously by Sanz-Leon et al. 2013 in a general overview of TVB.

### 1.2. Obtaining and using TVB

The easiest way to get started with TVB is to download a distribution^1^, which is available for Windows, Mac OS X and Linux. This distribution includes all of pieces, from the simulator to the web interface, and has no requirements other than a modern web browser supporting WebGL, SVG and HTML5.

Alternatively, because TVB is licensed under the GPL v. 2, the sources may be readily obtained from the public Git repositories hosted on GitHub^2^ (Dabbish et al., 2012). In order to use the simulator, only the standard scientific Python packages are required (NumPy and SciPy). The framework and web interface depend on a few more packages.

Documentation including an installation guide, web interface and console user guides as well as other information are available online^3^. We provide an API doc, built from the docs strings using Sphinx. User's, Contributor's and Developer's manuals are also provided with TVB distributions in PDF format. The source files (in.rst) are available from the Git repositories. In addition, IPython notebooks (Pérez and Granger, 2007) for interactive tutorials are provided. These are based on the demonstration scripts provided with the scientific library, and include a more detailed description of the scientific goal (if applicable), the components and stages of a simulation as well as a brief description in the case of reproducing previous work. Users interacting with *TheVirtualBrain* GUI may also benefit from these tutorials. Finally, the user interface provides an online help overlay, that pulls information from the User's manual.

## 2. Architecture

**Figure 1:**
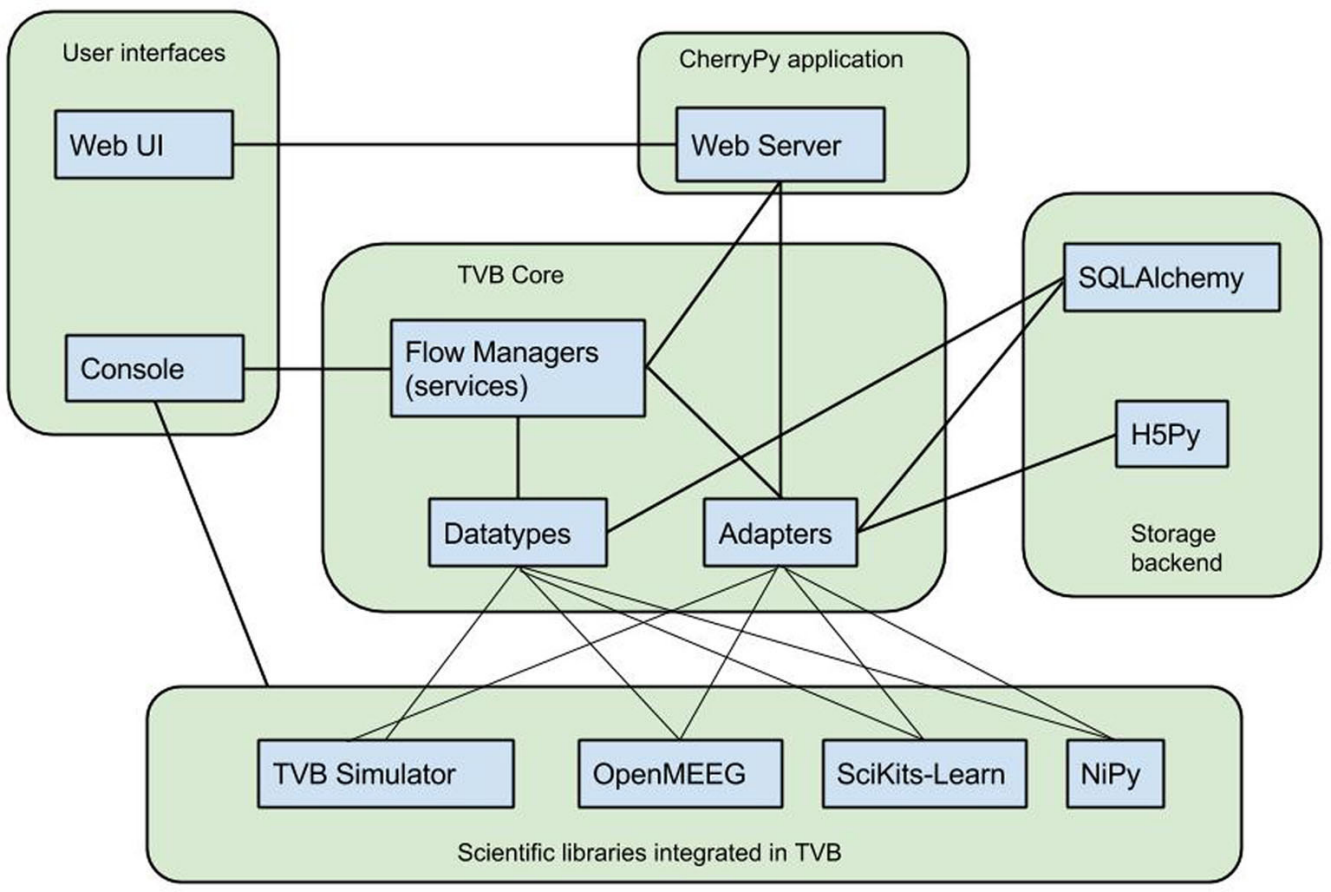
**TVB Architecture**. *TheVirtualBrain* integrates various scientific libraries through its flexible datatype and adapter abstractions, which allow web and console users to drive work flows in a generic way as well as persistence in a hybrid relation and HDF5 data store.

TVB's architecture, illustrated in Figure 1, was designed for the integration of disparate computational tools, allowing different kinds of data to be managed within one system, and different routines and processes to work with the kinds of data in the system. To facilitate this integration, two abstractions have been introduced, around which the framework is oriented: *datatypes* and *adapters*, serving, briefly, as heavily annotated structures and functions allowing for programmatic interoperation with the database and generation of and interaction with the user interface.

### 2.1. Datatypes

Concretely, a TVB datatype is a Python class with one or more attributes that are *trait*s, where a trait specifies both the kind of data expected for the corresponding attribute, as well additional metadata to aid in storage and user interface construction.

During the development of the datatype and traits system, existing implementations of the concepts of traits were considered, notably Enthought's extensive implementation designed to accelerate the design of graphical scientific applications and IPython's lighter-weight system designed, to a large extent, to provide a robust configuration system. In both cases, the traits are used to explicitly specify types, allowing runtime values' types to be validated, as well as other forms of introspection. This is the case for TVB's traits as well, however, Enthought's implementation has significant compiled extensions (where TVB is intended to be pure-Python); IPython's implementation does not provide support for arrays, and neither provides integration or mapping to a database. Lastly, Nipype (Gorgolewski et al., 2011) provides an approach specifically targeted toward neuroimaging, but is focused on data processing, whereas TVB required database and UI integration. For these reasons, it was judged useful to develop a system adapted to the needs of TVB.


**class** Point2D(MappedType):
    x = Float(label="x",
              doc="horizontal position of point")

    y = Float(label="y",
              doc="vertical position of point")


Code 1: Example of a simple datatype modeling a point in two dimensions.

Listing 1 shows a datatype modeling a point in two dimensions, consisting of two floating point values, x and y. This class derives from MappedType whose metaclass, before creating the class object, filters attributes for trait instances or types and creates a corresponding SQLAlchemy model, which results in mapping instances of Point2D to a corresponding table in TVB's SQL database. The trait base class implements a data descriptor protocol, i.e., __set__ in addition to __get__ methods, which, for a MappedType instance, forwards calls to the get and set methods to the corresponding SQLAlchemy method, in turn, interacting with the database.

When methods of such a class with annotated attributes are invoked, they may use the traited attributes directly, accessing either a default value or one given during the instantiation of the object. Additionally, this allows the web-based user interface to introspect a class for all of its attributes and their descriptions, to provide help and choose the proper display form. The explicit typing also allows such classes to be nearly automatically mapped to storage tables, providing persistence, when the storage layer is enabled. Lastly, because such metadata is used to build the docstring of a class, the console user also may obtain extensive descriptions of class, attributes, methods and arguments in the usual way. Table 1 lists the various parts of a traited attribute and how they are used.

**Table 1:** **TVB currently available traited attributes**.

**Keyword name**	**Description**
default	Default value for current attribute. Will be set on any new instance if not specified otherwise in the constructor
console_default	Define how a default value can be computed for current attribute, when console interface is enabled
range	Specify the set of accepted values for current attribute. Mark that this attribute is usable for parameter space exploration
label	Short text to be displayed in UI, in front of current attribute
doc	Longer description for current attribute. To be displayed in UI as help-text
required	Mark current attribute as required for when building a new instance of the parent class
locked	When present and *True*, current attribute will be displayed as read-only in the web interface
options	Used for attributes of type *Enumerate*, specifying the accepted options as a list of strings
filters_ui	SQL filters on other attributes, to be applied in UI
select_multiple	When *True*, current attribute will be displayed as a select with multiple options in UI (default is single-select)
order	Optional number identifying the index at which current attribute will be displayed in UI
	When negative, the attribute is not displayed at all. Ascending order for indices is considered when displaying
use_storage	When *False*, current attribute is not stored in database or file storage
file_storage	Valid values for this attribute are: *None*, *HDF5*, or *expandable_HDF5*,
	When *None*, current attribute is not stored in the file-storage at all. When *HDF5*, we use regular HDF5 file storage
	When *expandable_HDF5* value is set, a HDF5 stored in chunks is used

Several trait types are built into TVB's traits system, such as dictionaries, tuples, lists and arrays, and in most cases, a string representation of the trait value is stored in the database. Persisting arrays in SQL, however, is relatively inefficient, and for this case the data are automatically stored in HDF5 files. Such options as well as fine-tuning the presentation of different traits in the user interface are also specified by keyword arguments to the trait specification. Table 1 describes several of these keywords used throughout the various datatypes in TVB.

In TVB, the datatype classes must typically implement significant functionality with respect to the user interface, such as filtering instances based on a user's criterion, as well as scientific methods, such as computing the geodesic distance on a surface. To facilitate the organization of the code, a base class declaring the traited attributes is created, followed by two subclasses for framework and scientific methods, and a final class that uses the framework and scientific classes as mixins. The advantage of this scheme is that the domain data models can be grouped, and the framework and scientific code may be separated.

### 2.2. Adapters

The adapter pattern allows arbitrary processes to be invoked by a generic framework by detailing the required input data, the outputs, and providing a method for invoking the process. The input and output data are described in terms of TVB's datatypes, which allows processes with different data formats to interact as long as an intermediate, common datatype is available. In TVB, this abstraction extends not only to computational processes such as simulation and analysis, but data import, export and, notably visualizations.


class DistanceAdapter(ABCAdapter):

**def** get_input_tree(self):
    **return** 'p': Point2D(), 'q': Point2D()

**def** get_output(self):
    **return** [Float]

**def** launch(self, x, y, sqrt=math.sqrt):
    **return** sqrt((p.x - q.x)**2 + (p.y - q.y)**2)


Code 2: Example of a minimal adapter.

Listing 2 presents a minimal example that computes the distance between two points. Descriptions of the inputs and outputs are provided by the get_input_tree and get_output methods, and the computation itself is set off by the method. In practice, additional methods are used on adapters to provide for more complex initialization and to obtain more information about the space and time requirements of the computation.

This approach scales up to more complex computations, and notably, the simulator itself is integrated with the web interface through via a SimulatorAdapter. Additionally, due to the wide-variety of toolboxes available for the MATLAB environment, an adapter was created to allow arbitrary code to be called on any given data type. This adapter works by filling out a template driver script to handling loading and saving data and launching the desired code within a try-except clause. This script is with MATLAB or Octave via a call to the os.system function.

Several alternatives to this approach are possible, such as invoking the MATLAB engine directly via ctypes and the MATLAB Engine API, or compiling the MATLAB functions with the MATLAB Compiler. Each, including our approach, has advantages and disadvantages. In our case, when configuring TVB, the administrator is asked to provide the path to the MATLAB or Octave executable, and TVB attempts to verify that this executable can be invoked. Beyond this, no other verification or protection is currently provided against problematic situations, in part because we have found it to be sufficiently robust.

Launching MATLAB can be a relatively slow operation (cold startup 8 s, cached 1 s; on a Linux workstation), and where Octave is available, it is faster (0.1 s). For our use cases, e.g., launching analyses, this works without problems in a single user situation. In the case that many operations are necessary, they can be batched into the same script such that MATLAB is called but once.

One of the uses of this adapter, employs a well-known toolbox for characterizing anatomical and functional connectivity, the *Brain Connectivity Toolbox* (Rubinov and Sporns, 2010). In TVB, the set of functions, their inputs and outputs are summarized in an XML file, which is read by a generic adapter, which handles the data appropriately.

#### 2.2.1. Analysis and visualizers

TVB does not intend to provide fully featured, complex data analysis techniques, which have been well covered by other packages. Instead, we offer a minimal set of standard algorithms to quickly validate simulation results or compare with imported patient data; these include principal and independent component analyses, Fourier and wavelet spectral analyses, correlation and coherence analyses.

TVB visualizers employ different techniques, depending on the nature of the data to display and degree of interactivity required, including WebGL, D3.js generated SVG, Matplotlib's HTML5 Canvas backend, as well as other HTML5 Canvas Javascript libraries for simpler, static graphs. In each case, an adapter is developed to abstract over the differences between these techniques, allowing the framework to treat it without knowing the details.

Interaction-intensive tools that combine several techniques have been developed for specific purposes, for example working with connectivity matrices, configuring the phase space structure of the mass model, or designing spatiotemporal stimuli. These are built upon the same abstractions and have been detailed in the general overview of TVB (Sanz-Leon et al., 2013).

## 3. Simulator

A significant part of TVB is simulating large-scale brain networks. While several existing simulators could have been adapted, we have estimated that TVB style simulations are far enough outside the design of other simulators to make a new development necessary. We discuss these reasons in the following.

Existing neural network simulators typically focus either on abstract rate neurons, modeling neurocognitive processes, or multicompartmental neurons treating complex spatial geometries, e.g., NEURON (Hines and Carnevale, 2001), modeling the interaction of channel distributions in dendrites. More recently, due to interest in the computational properties of spiking neurons and their relevance to experimental observations, simulators designed for spiking or oscillating neurons have become prominent, including Brian (Goodman and Brette, 2009), which we initially considered for our simulations. In TVB the network is defined with neural mass or field models (Deco et al., 2008; Coombes, 2010) rather than cellular models. The spatial extent of the modeled dynamics is macroscopic and scales reasonably to the entire cortex, and uses empirical measurements of cortico-cortical connectivity. Several technical issues are unique to this scale, such as efficient handling of dense *N*^2^ inter-regional delays and integration of neural field-like models and connectivity on triangular meshes in 3D. Finally, comparison with experimental data requires forward solutions that transform physiological signals to the commonly used imaging modalities such as EEG, MEG and fMRI. For these reasons, TVB required a new simulator, built around the paradigm of whole-brain scale simulation.

The simulator in TVB resembles popular neural network simulators in many fundamental ways, both mathematically and in terms of informatics structures, however, we have found it necessary to introduce auxiliary concepts particularly useful in the modeling of large scale brain networks. In the following, we will highlight some of the interesting principles and capabilities of TVB's simulator and give rough characterization of the execution time and memory required in typical simulations.

### 3.1. Node dynamics

In TVB, nodes are not considered to be abstract neurons nor necessarily small groups thereof, but rather large populations of neurons. Concretely, the main assumption of the neural mass modeling approach in TVB is that large pools of neurons on the millimeter scale are strongly approximated by population level equations describing the major statistical modes of neural dynamics (Freeman, 1975). Such an approach is certainly not new; one of the early examples of this approach consist of the well known Wilson–Cowan equations (Wilson and Cowan, 1973). Nevertheless, there are important differences in the assumptions and goals from modeling of individual neurons, where the goal may be to reproduce correct spike timing or predict the effect of a specific neurotransmitter. A second difference lies in coupling: chemical coupling is often assumed to be pulsatile, or discrete, between neurons, whereas for mesoscopic models it is considered continuous. Typically the goal of neural mass modeling is to study the dynamics that emerge from the interaction of two or more neural masses and the network conditions required for stability of a particular spatiotemporal pattern. In the following, we shall briefly discuss some of the models available in TVB.

This modeling paradigm may be contrasted with population density models modeling the dynamics of large populations of neurons (De Groff et al., 1993; Knight, 2000; Omurtag et al., 2000; Gerstner, 2000; see Deco et al., 2008 for a review). The large number of neurons impart the state space with a probability density, and population density methods describe the evolution of this density via the Fokker–Planck (FP) equation (Risken, 1996) comprised of flow and dispersion terms. This approach assumes that the afferents on neurons in one population are uncorrelated. Neural mass models, from this paradigm, are obtained as a special case when the full ensemble density dynamics is replaced by a mass at one point in state space and its dynamics summarize the movement of density in state space, losing information on the changes in shape of the density. In the full, non-linear FP form, different density moments can couple, even within and between populations, meaning the membrane potential variance of one population could affect the average of another. Neural mass models ignore this possibility by coupling only the first moments, though this may be overcome by e.g., extending mass models with more than one mode (Stefanescu and Jirsa, 2008, 2011). TVB does not implement density methods via the FP equation because the additional moments required to derive physiologically relevant variables (such as LFP or firing rate), would add an additional level of complexity.

In TVB, many neural mass models from the literature are available, including the often used Jansen–Rit model of rhythms and evoked responses arising from coupled cortical columns (Zetterberg et al., 1978; Jansen and Rit, 1995; Spiegler et al., 2010). David and Friston's slightly modified form has been extensively applied within a Bayesian framework known as Dynamic Causal Modeling (DCM) for modeling neuroimaging data via estimation of biophysical parameters of underlying network models (David and Friston, 2003; Friston et al., 2003; David et al., 2006). The Jansen–Rit model is a biophysical one, whose state variables and parameters are readily interpretable with respect to experiments, however, it has at least six state equations involving several exponential expressions. For cases where it is desirable to have a simpler and more performant model, a generic two-dimensional oscillator model is also provided by TVB [see Strogatz (2001) and Guckenheimer and Holmes (1983) for for generic mathematical references on two-dimensional dynamical systems]. This model produces damped, spike-like or sinusoidal oscillations, which, in the context of a network, permit the study of network phenomena, such as synchronization of rhythms or propagation of evoked potentials. Other models implemented in TVB include the Wilson–Cowan description of functional dynamics of neural tissue (Wilson and Cowan, 1972), the Kuramoto model describing synchronization (Kuramoto, 1975; Cabral et al., 2011), two and three dimensional statistical mode-level models describing populations with excitability distributions (Stefanescu and Jirsa, 2008, 2011), a reduction of Wong and Wang's (2006) model as presented by Deco et al. (2013) and a lumped version of Liley's model (Liley et al., 1999; Steyn-Ross et al., 1999). Lastly, adding a model only requires subclassing a base Model class and providing a dfun method to compute the right hand sides of the differential equations.

### 3.2. Network structure

The network of neural masses in TVB simulations directly follows from two geometrical constraints on cortical dynamics. The first is the large-scale white matter fibers that form a non-local and heterogeneous (translation variant) connectivity, either measured by anatomical tracing (CoCoMac, Kötter, 2004) or diffusion-weighted imaging (Hagmann et al., 2008; Honey et al., 2009; Bastiani et al., 2012). The second is that of horizontal projections along the surface, which are often modeled with a translation invariant connectivity kernel, approximating a neural field (however, as with other parameters in the simulator, connectivity kernel parameters that vary across space can also be used).

TVB does not assume that the network structure is constant during the simulation, but does not currently implement the modeling of structural modulation. This can, however, be added during simulation.

#### 3.2.1. Large-scale connectivity

The large-scale region level connectivity at the scale of centimeters, resembles more a traditional neural network than a neural field, in that, neural space is discrete, each node corresponding to a neuroanatomical region of interest, such as V1, etc. It is at this level that inter-regional time delays play a large role, whereas the time delays due to lateral, local projections are subsumed under the dynamics of the node.

It is often seen in the literature that the inter-node coupling functions *are* part of the node model itself. In TVB, we have instead chosen to factor such models into the intrinsic neural mass dynamics, where each neural mass's equations specify how connectivity contributes to the node dynamics, and the coupling function, which specifies how the activity from each region is mapped through the connectivity matrix. Common coupling functions are provided such as the linear, difference and periodic functions often used in the literature.

#### 3.2.2. Local connectivity

The local connectivity of the cortex at the scale of millimeters provides a continuous 2D surface along horizontal projections connecting cortical columns. Such a structure has previously been modeled by neural fields (Amari, 1977; Jirsa and Haken, 1996, 1997; Liley et al., 1999). In TVB, surface meshes provide the spatial discretization of neural anatomy. A neural mass is placed on each vertex, and the geodesic distances between each mass is computed. A local connectivity kernel assigns, for each distance, a connection weight between masses. This kernel is usually chosen such that it decays exponentially with distance. The associated sampling problem has been studied in detail by Spiegler and Jirsa (2013), which finds the approximation to be reasonable, depending on the properties of the mesh and the imaging modalities that sample the activity simulated on the mesh. In fact, the implementation of the local connectivity kernel is such that is can be re-purposed as a discrete Laplace–Beltrami operator, allowing for the implementation of true neural field models that use a second-order spatial derivative as their explicit spatial term.

TVB currently provides several connectivity kernels, of which a Gaussian is one commonly used. Once a cortical surface mesh and connectivity kernel and its parameters are chosen, the geodesic distance (i.e., the distance along the cortical surface) is evaluated between all neural masses (Mitchell et al., 1987), and a cutoff is chosen past which the kernel falls to 0. This results in a sparse matrix that is used during integration to implement the approximate neural field.

### 3.3. Integration of stochastic delay differential equations

In order to obtain numerical approximations of the network model described above, TVB provides both deterministic and stochastic Euler and Heun integrators, following standard numerical solutions to stochastic differential equations (Mannella and Palleschi, 1989; Klöden and Platen, 1995; Mannella, 2002).

While the literature on numerical treatment of delayed or stochastic systems exists, it is less well known how to treat the presence of both. For the moment, the methods implemented by TVB treat stochastic integration separately from delays. This separation coincides with a modeling assumption that in TVB the dynamical phenomena to be studied are largely determined by the interaction of the network structure and neural mass dynamics, and that stochastic fluctuations do not fundamentally reorganize the solutions of the system (Ghosh et al., 2008; Deco et al., 2009, 2011, 2012).

Due to such a separation, the implementation of delays in the regional coupling is performed outside the integration step, by indexing a circular buffer containing the recent simulation history, and providing a matrix of delayed state data to the network of neural masses. While the number of pairwise connections rises with *n*^2^_*region*_, where *n*_*region*_ is the number of regions in the large-scale connectivity, a single buffer is used, with a shape (*horizon, n_cvar_, n_region_*) where *horizon* = *max(delay)* + 1, and *n*_*cvar*_ is the number of coupling variables. Such a scheme helps lower the memory requirements of integrating the delay equations.

### 3.4. Forward solutions

TVB provides forward solutions to generate neuroimaging data from simulated neural activity based on biophysical models (Jirsa et al., 2002; Buxton et al., 2004). Practically, it is also often necessary to simply reduce the size of data, especially in the case of surface simulations. TVB implements these two functionalities in a set of classes called Monitors, each of which receives the raw simulation output and applies a spatial, temporal or spatiotemporal kernel to the output to obtain the simulation output passed to the user or stored.

Where necessary, monitors employ more than one internal buffer. In the case of the fMRI monitor, a typical sampling frequency of simulation may be upward of 64 kHz, and the haemodynamic response function may last several seconds, using only a single buffer could require several gigabytes of memory for the fMRI monitor alone. Given that the time-scale of simulation and fMRI differ by several orders of magnitude, the subsequent averaging and downsampling is justified.

In the cases of the EEG and MEG monitors, the temporal kernel implements a simple temporal average, and the spatial kernel consists of a so-called lead-field matrix as typically derived from a combination of structural imaging data, providing the locations and orientations of the neural sources and the locations and orientations of the EEG electrodes and MEG gradiometers and magnetometers. As the development and implementation of such lead-fields is well developed elsewhere (Sarvas, 1987; Hamalainen and Sarvas, 1989; Jirsa et al., 2002; Nolte, 2003; Gramfort et al., 2010), TVB provides access to the well-known OpenMEEG package.

### 3.5. Parameter sweeps

As is often the case in modeling, there are several or many parameters of the simulation that may be relevant, and to facilitate the exploration of the effects of variation of parameters, the user, from the web interface, can select one or two parameters and create a grid of simulations that are run in parallel on the computer. Afterwards, a summary visualization of the simulations is displayed. For example, this simple approach can be useful to find the parameter values within a range nearest to a bifurcation from a fixed point to a limit cycle: to each resulting time series, the global variance is computed, and displayed as a function of parameter values.

Nevertheless, no tools are provided to perform correct estimation or fitting of the models, and this is not currently a goal for TVB.

For this purpose, as well as more sophisticated explorations of the parameter space it may be useful to turn to scripting the simulator in Python, and pulling in functionality from other libraries.

### 3.6. Performance

A priority of the simulator in TVB is to attain a high level of performance while remaining in pure Python. In order to see where the simulator spends most of its time, we have profiled a set of eight characteristic simulations on function timing as measured by the cProfile module of the standard library.

Measurements were performed on an HP Z420 workstation, with a single Xeon E5-1650 six-core CPU running at 3.20 GHz, L1-3 cache sizes 384, 1536, and 12 MB, respectively, with main memory 4 × 4 GB DDR3 at 1600 Mhz, running Debian 7.0, with Linux kernel version 3.2.0-4-amd64. The 64-bit Anaconda Python distribution was used with additional Accelerate package which provides acceleration of common routines based on the Intel Math Kernel Library. A Git checkout of the trunk branch of TVB was used with SHA 6c644ab3b5.

Eight different simulations were performed corresponding to the combinations of either the generic 2D oscillator or Jansen–Rit model, region-only or use of cortical surface, and two conduction speeds, *v_c_* = 2.0 and *v_c_* = 20.0 (m/s). In each case, a temporal average monitors at 512 Hz is used, and the results are discarded. The region-only simulation was run for 1 s while the surface simulation was run for 100 ms.

As can be seen in Table 2, significant time is spent on array manipulations written in C (marked by those function names surrounded by angle brackets), from the NumPy and SciPy libraries, including the spare matrix-vector multiplication used to compute the local connectivity. In some cases, such as the neural mass model definitions, large expressions implicitly generate many temporary arrays, which can be ameliorated by using the numexpr module to compute the expressions element-wise: TVB's JansenRit model is implemented as regular Python expressions of NumPy arrays, while a subclass JRFast uses numexpr.evaluate, resulting in a 40% improvement in overall runtime.

**Table 2:** **Profiling results for several simulation types, “R” for region level simulations, “S” for surface level**.

**Sim**.	**Time (s)**	**Module/function**
R / G2D / 20	11.72	<numpy.core.multiarray.array>
	6.14	numpy.lib.npyio, loadtxt
	5.18	tvb.simulator.simulator, __call__
	3.18	numpy.lib.npyio, pack_items
	2.56	numexpr.necompiler, evaluate
2	11.87	<numpy.core.multiarray.array>
	6.10	numpy.lib.npyio, loadtxt
	5.54	tvb.simulator.simulator, __call__
	3.16	numpy.lib.npyio, pack_items
	2.50	numexpr.necompiler, evaluate
JR / 20	14.21	<numpy.core.multiarray.array>
	9.99	tvb.simulator.simulator, __call__
	7.28	tvb.simulator.models, dfun
	6.20	numpy.lib.npyio, loadtxt
	3.24	numpy.lib.npyio, pack_items
2	14.21	<numpy.core.multiarray.array>
	10.57	tvb.simulator.simulator, __call__
	7.40	tvb.simulator.models, dfun
	6.12	numpy.lib.npyio, loadtxt
	3.25	numpy.lib.npyio, pack_items
S / G2D / 20	126.61	<_csc.csc_matvec>
	57.56	<numpy.core.multiarray.array>
	56.17	<gdist.local_gdist_matrix>
	9.05	<numpy.core._dotblas.dot>
	7.56	numpy.lib.npyio, loadtxt
2	125.95	<_csc.csc_matvec>
	57.75	<numpy.core.multiarray.array>
	56.16	<gdist.local_gdist_matrix>
	12.10	<numpy.core._dotblas.dot>
	7.37	numpy.lib.npyio, loadtxt
JR / 20	126.31	<numpy.core.multiarray.array>
	56.37	<gdist.local_gdist_matrix>
	19.52	<numpy.core._dotblas.dot>
	9.47	tvb.simulator.models, dfun
	8.87	<mtrand.RandomState.normal>
2	126.09	<numpy.core.multiarray.array>
	56.79	<gdist.local_gdist_matrix>
	29.10	<numpy.core._dotblas.dot>
	14.18	<mtrand.RandomState.normal>
	9.57	tvb.simulator.models, dfun

*“G2D” signifies the generic two-dimensional oscillator whereas “JR” is the Jansen–Rit model. Finally, either 20 m/s or 2 m/s conduction velocity was used. Time is given as the total time spent in the method or function listed in the right column*.

Given that the numerical routines are written in Python to maintain a high level of flexibility, it is expected that there are limits on the performance, especially compared to compiled languages. Ongoing work on the simulator will take advantage of newer programming paradigms and hardware, such as OpenCL and graphics processing units; this sort of programming is facilitated by Python libraries such as PyOpenCL (Klöckner et al., 2012).

## 4. Discussion

In this article, we have described several informatics aspects of the implementation of a integrative platform for modeling neuroimaging data. Despite the currently available functionality in TVB, in the following we wish to make several points about its context as a modeling tool as well as future work.

### 4.1. Modeling goals

The literature on network models frequently presents work in which a model is constructed in order to estimate structure and parameters from experimental data, and the DCM framework has significantly advanced methods for such estimations for both fMRI and M/EEG data. Indeed, there are similarities in the underlying mathematical machinery between DCM and TVB. However, the estimation of parameters in the case of TVB's models is still a challenging question and for now is not a goal of the framework. For this reason, none of the requisite estimation tools are currently provided by TVB.

Related to the goal of estimating model parameters for brain network models is the modeling of function or functional dynamics itself (Erlhagen and Schöner, 2002; Eliasmith et al., 2012). TVB allows a user to fully specify the node dynamics and connectivity, yet no support is currently providing for deriving a set of parameters that leads to a particular functional state. This, like the estimation problem, is an open question, particularly for models as complex as those simulated within TVB.

### 4.2. Future work

Since the recent release of the 1.0 version of TVB, it has been officially considered *feature* complete, however, in several cases, the development of features has outstripped documentation and testing. Going forward, general priorities include advancing test coverage, improving documentation for users, and preparing PyPI and Debian packages. In the mean time, TVB's Google groups mailing list continues to provide an open forum for troubleshooting and sharing of user experiences.

While several mathematical challenges are presented by the TVB models, one of the bottlenecks is the speed of the numerical simulation. To address this, continued optimization of C and GPU code generation will take place, e.g., to perform parallel parameter sweeps.

Additionally, an interface *from* MATLAB to TVB is being developed to allow use of the simulator through a simple set of MATLAB functions. As this infrastructure is based on an HTTP and JSON API, it will likely enable other applications to work with TVB as well.

Lastly, as TVB was originally motivated to allow a user to move from acquired data to simulated data as easily as possible, we will continue to integrate several of the requisite steps that are not currently covered, such as analysis of DSI data to produce connectivity matrices, though in many cases, such as parcellation and labeling of cortical areas, these steps may continue to require interaction with other software. Altogether, TVB provides a rich platform for integrating neuroinformatics tools with an emphasis on analysis and modeling of human neuroimaging data.

### Conflict of interest statement

The authors declare that the research was conducted in the absence of any commercial or financial relationships that could be construed as a potential conflict of interest.

## Footnotes

^1^http://www.thevirtualbrain.org

^2^https://github.com/the-virtual-brain

^3^http://the-virtual-brain.github.io
